# Integrated Profiling of Fatty Acids, Sterols and Phenolic Compounds in Tree and Herbaceous Peony Seed Oils: Marker Screening for New Resources of Vegetable Oil

**DOI:** 10.3390/foods9060770

**Published:** 2020-06-11

**Authors:** Xiaoqin Wang, Chunhuan Li, María del Mar Contreras, Vito Verardo, Ana María Gómez-Caravaca, Chen Xing

**Affiliations:** 1 College of Chemical Engineering, Huaqiao University, Xiamen 361021, China; 18013087011@stu.hqu.edu.cn (C.L.); 18013087010@stu.hqu.edu.cn (C.X.); 2 Institute of Oil and Natural Product, Huaqiao University, Xiamen 361021, China; 3 Shandong Institute for Food and Drug Control, Jinan 250101, China; 4 Department of Chemical, Environmental and Materials Engineering, Universidad de Jaén, Campus Las Lagunillas, 23071 Jaén, Spain; 5 Department of Nutrition and Food Science, University of Granada, Campus of Cartuja, 18071 Granada, Spain; vitoverardo@ugr.es; 6 Institute of Nutrition and Food Technology ‘José Mataix’, Biomedical Research Centre, University of Granada, Avenida del Conocimiento s/n, E-18071 Granada, Spain; 7 Department of Analytical Chemistry, University of Granada, c/Fuentenueva s/n, E-18071 Granada, Spain; anagomez@ugr.es

**Keywords:** tree peony seed oil, fatty acids, sterols, phenolic compounds, UHPLC-Q-TOF-MS/MS

## Abstract

Tree peonies (*Paeonia ostii* and *Paeonia rockii*) are popular ornamental plants. Moreover, these plants have become oil crops in recent years. However, there are limited compositional studies focused on fatty acids. Therefore, this work aims to reveal compositional characteristics, regarding fatty acids, sterols, γ-tocopherol and phenolic compounds, of tree peony seed oils from all major cultivation areas in China, and to compare with herbaceous peony seed oil. For that, an integrative analysis was performed by GC-FID, GC-MS and UHPLC-Q-TOF-MS technologies. The main fatty acid was α-linolenic acid (39.0–48.3%), while β-sitosterol (1802.5–2793.7 mg/kg) and fucosterol (682.2–1225.1 mg/kg) were the dominant phytosterols. Importantly, 34 phenolic compounds, including paeonol and “*Paeonia* glycosides” (36.62–103.17 μg/g), were characterized in vegetable oils for the first time. Conclusively, this work gives new insights into the phytochemical composition of peony seed oil and reveals the presence of bioactive compounds, including “*Paeonia* glycosides”.

## 1. Introduction

Tree peonies originate from China and they have become popular ornamental plants all over the world. In addition, they are used to produce an important Chinese medicine, termed as Moutan Cortex, from the root bark. Recently, two tree peony species, *Paeonia ostii* T.Hong and J.X.Zhang and *Paeonia rockii* (S.G.Haw and Lauener) T.Hong and J.J.Li ex D.Y.Hong arouse the attention of oil producers because they are simple flowers with large outputs of seeds, which contain up to 30% oil. Interestingly, the seed oils have a strong fragrance of tree peony flowers. Most importantly, their seed oil contains more than 38.7% α-linolenic acid and has become a new resource of vegetable oil with functional properties [1].

At present, the research has focused on the analysis of fatty acid in tree peony seed oils [2,3,4]. Another category of important nonpolar compounds, phytosterols, has not been reported in tree peony seed oil to the best of our knowledge. The analysis of fatty acids and sterols is necessary as the basis of the evaluation of the quality of vegetable oil, according to the Codex Alimentarius.

As mentioned above, *P. ostii* and *P. rockii* also produce Moutan Cortex, containing plenty of active constituents; Paeonol 20.61 mg/g and paeoniflorin 11.44 mg/g were reported in Moutan Cortex [5]. Using high-performance liquid chromatography (HPLC) coupled with quadrupole-time-of-flight (Q-TOF)-mass spectrometry (MS), 5 primary metabolites and 41 secondary ones were identified in the root bark of tree peony [6]. Due to the same source, the flowers and seeds of the tree peony also contained phenolic compounds [7,8]. Even the extraction of monoterpene glycoside from the oil-extracted residues of tree peony seeds was investigated [9]. As for tree peony seed oil, there is no report about the phenolic compounds at present.

In the genus *Paeonia*, the *Paeonia* section, which includes *Paeonia lactiflora* Pall., also attracts the attention of oil-producers, and little comparative analysis has reported on the composition of tree and herbaceous peony seed oils, this being confused. Obviously, the comprehensive characterization of tree peony seed oil in comparison with herbaceous peony seed oil will promote the development of *Paeonia* plants as oil crops, supplying a clear qualitative and quantitative definition through compositional analysis. Thus, after the investigation into the growing areas of *P. ostii* and *P. rockii*, 10 collecting sites in China were selected. In addition, two varieties of the herbaceous peony with relatively high production of seeds were also included, to be compared with the tree peony. In this work, the fatty acids, phytosterols and phenolic compounds in *Paeonia* seed oils were analyzed with gas chromatography (GC)-flame ionization detection (FID), GC-MS and with ultra-HPLC-Q-TOF-MS/MS technology, respectively, and this work characterized bioactive “*Paeonia* glycosides” in vegetable oils for the first time.

## 2. Materials and Methods

### 2.1. Plant Materials

**Figure 1:**
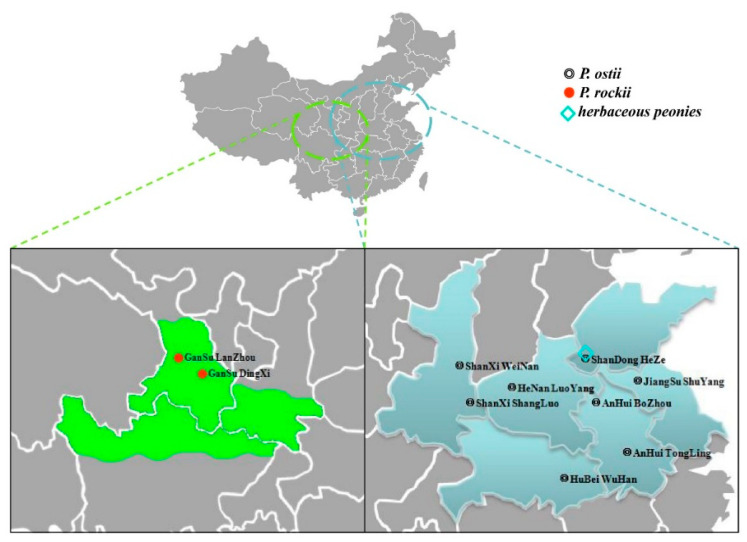
The sketch map of *P. ostii* and *P. rockii* distribution and the collecting sites of tree peony and herbaceous peony seeds in this work. GanSuLanZhou (GSLZ); GanSuDingXi (GSDX); ShanXiWeiNan (SXWN); ShanXiShangLuo (SXSL); HeNanLuoYang (HNLY); ShanDongHeZe (SDHZ); JiangSuShuYang (JSSY); AnHuiBoZhou (AHBZ); AnHuiTongLing (AHTL); HuBeiWuHan (HBWH).

Tree peony seeds were sampled from 10 main growing regions in China, including 8 *P. ostii* samples and 2 *P. rockii* samples: GanSuLanZhou (GSLZ); GanSuDingXi (GSDX); ShanXiWeiNan (SXWN); ShanXiShangLuo (SXSL); HeNanLuoYang (HNLY); ShanDongHeZe (SDHZ); JiangSuShuYang (JSSY); AnHuiBoZhou (AHBZ); AnHuiTongLing (AHTL); HuBeiWuHan (HBWH). In addition, two cultivars of herbaceous peony were procured, *P. lactiflora* ‘Tuopanxianbao’ and ‘Liantai’, from SDHZ (Figure 1). The mature seeds were harvested in early autumn (from August to the beginning of September, depending on the latitude of the collection sites) with random sampling, and mixed evenly in each site. Then, these seeds were naturally dried and stored in a shady and cool place. After being unshelled, some seeds were grounded with a high-speed pulverizer for Soxhlet extraction with *n*-hexane. The seed oils were extracted using a Soxhlet extractor for fatty acid and sterol analysis. The oil content of the analyzed tree peony seeds was from 27.7% to 34.0%, similar to that of the two herbaceous peony seeds, i.e., 26.9% and 32.2%, respectively (Table S1). For phenolic compound analysis, the seeds were crushed using screw press to obtain oil, according to the methods reported [10,11].

### 2.2. Standards and Reagents

A certified fatty acids methyl ester (FAME) reference standard mixture (37 fatty acids from C4 to C24) was obtained from Sigma-Aldrich (St. Louis, MO, USA). The internal standard 5α-Cholestan-3β-ol (purity ≥ 95%) was purchased from Sigma-Aldrich. The other reagents were supplied by Sinopharm (Shanghai, China). The solvents, *n*-hexane, methanol and acetonitrile, were of HPLC grade (Sigma-Aldrich). The analytical standards, including benzoic acid, cinnamic acid, *p*-hydroxyphenylacetic acid, naringin, paeoniflorin, paeonol and oxyresveratrol, were purchased from Aladdin Co., Ltd. (Shanghai, China).

### 2.3. Gas Chromatography (GC) Analysis of Fatty Acids

Fatty acids (FAs) were determined as methyl-esters according to previous study [10], with an Agilent 6890N gas chromatograph (Agilent Technologies, Santa Clara, CA, USA) equipped with an DB-23 capillary column (60 m × 0.25 mm × 0.25 μm; Agilent Technologies), a split injector and a flame ionization detector at 270 and 250 °C, respectively. The samples prepared were injected at a split ratio of 20:1. The programmed temperature was increased from 130 to 170 °C at a rate of 6.5 °C/min, and then increased to 215 °C at a rate of 2.75 °C/min and held for 12 min, finally increased to 230 °C at a rate of 40 °C/min and held for 0.5 min. The identification of the chromatographic peaks was performed by comparing the retention time of sample with a certified FAME mix and quantitative analysis by area normalization method.

### 2.4. GC-MS Analysis of Squalene, γ-Tocopherol and Phytosterols

Squalene, γ-tocopherol and phytosterols were analyzed as reported [10]. After pre-treatment, 1 μL of product was analyzed by GC-MS. The extracts were analyzed by a Shimadzu GC-MS QP-2010 Plus (Shimadzu Corp, Kyoto, Japan) equipped with a Rtx-5MS capillary column (30 m × 0.25 mm i.d. 0.15 μm film; J&W Scientific, Folsom, CA, USA). The carrier gas was helium with a flow rate of 1.3 mL/min; split ratio was 15:1. Analyses were performed under the following temperature program: oven temperature from 180 to 250 °C at a rate of 12 °C /min, and it was subsequently increased by 2 °C/min to 265 °C and kept isothermal for 22 min. Ion source temperature of the instrument was 300 °C, transfer line of 300 °C, and solvent delay was 7 min. Scan time and mass range were 0.5 s and 40–600 (*m/z*), respectively. Identification of sterols were achieved by a mass spectra database search (NIST05 Library) and co-eluted with corresponding standards.

### 2.5. UHPLC-Q-TOF-MS and -MS/MS Analysis of Phenolic Compounds

The phenolic compounds were extracted basically according to the report by Wang et al. [11] as follows. 2.5 g of oil was weighed and then mixed with 5 mL of *n*-hexane. The mixture was added with 6 mL of methanol–water (60:40, *v/v*) and was stirred for 3 min. After centrifugation at 3500× *g* for 10 min, the methanol phase was separated. The procedures above were repeated three times. All the methanolic fractions were combined to be evaporated to dryness at 35 °C and the residue was redissolved in 250 μL methanol–water (50:50, *v/v*). Finally, the solution was filtered through 0.2 μm organic membrane filters. The phenolic compounds were identified using HPLC (Agilent 1290 Infinity II, Agilent Technologies) coupled to a 6545 Q-TOF mass spectrometer (Agilent Technologies). Separation was achieved on an Agilent ZORBAX Eclipse Plus-C18 column (2.1 × 50 mm, 1.8 μm), which was thermostated at 30 °C, at a flow rate of 0.4 mL/min, using 2 μL of injection volume. The mobile phase was composed of 0.1% acetic acid in water (A) and 0.1% acetic acid in acetonitrile (B). The gradient elution involved a four-step program: 0–3 min, 5–10% B; 3–4 min, 10–20% B; 4–8 min, 20–55% B; 8–10 min, 55–90% B. A post-run of 3 min was programmed to equilibrate the column between analyses.

The electrospray ionization (ESI) source was operating in the negative ionization mode. The mass spectrometer parameters were as follows: capillary 4000 V; nebulizer pressure, 30 psi; fragment voltage, 140 V; drying gas flow rate, 9 L/min; gas temperature, 190 °C; N_2_ sheath gas temperature, 350 °C; N_2_ sheath gas flow rate, 10 L/min. The accurate mass spectra were recorded across the range of *m/z* 100–1000 in full scan mode. To assure the desired mass accuracy of the recorded ions, continuous internal calibration was performed during analyses with the use of signals at *m/z* 112.9855, 980.0163 and 1033.9881. Optimal ionization conditions were evaluated by a tuning fluid and the instrument provided a resolution of at least 100,000. Fragmentation was performed with the collision energy 10–40 eV. For the qualitative analysis, an in-house database of phenolic compounds from *Paeonia* and other relative plants was set up by our research team on the basis of the literature and databases, including ChemSpider, Pubmed, Scifinder, the Chemistry database of CAS (Chinese Academy of Sciences), etc. The quantitative analysis was performed by the determination of the peak area and according to the standard curves.

### 2.6. Statistical Analysis

All quantitative analyses were done in triplicate for each sample. Results shown were mean ± standard deviation (m ± SD). Statistical significance was examined through one-way analysis of variance and Duncan’s test at *p* ≤ 0.05 using the software SPSS 20.0. Hierarchical clustering analysis (HCA) was also performed with the software SPSS 20.0.

## 3. Results

### 3.1. Fatty Acids in Paeonia Seed Oils

The composition of the major fatty acids in all the investigated samples is shown in Table 1. Moreover, Table S1 also depicts minor ones. For the tree peony seed oils, the dominant fatty acids were α-linolenic acid (39.0–48.3%), which was much higher than all the named vegetable oils listed in CODEX STAN 210–1999. It was followed by linoleic acid (16.9–29.9%), oleic acid (20.6–26.8%), palmitic acid (4.9–5.9%) and stearic acid (1.6–2.3%), which accounted for 98.5–98.6% of the total fatty acids. It may be worth noting that the α-linolenic acid of *P. rockii* was 46.2–48.3%, higher than that of *P. ostii*, which was 39.0–44.9%.

Similarly, the percentage of α-linolenic acid in *P. ostii* and *P. rockii*, cultivated in a garden in Beijing, was 39.6% and 49.9%, respectively [12]. However, in other work, α-linolenic acid varied from 25.2% to 46.7% in the oils of *P. ostii* seeds [13]. Meanwhile, α-linolenic acid ranged between 26.7% and 49.7% in the seed oils of *Paeonia* plants [14].

As for the herbaceous peonies, the basic composition of fatty acids was similar to that of the tree peonies. α-Linolenic acid concentration was up to 43.7% in the seed oil of ‘Tuopanxianbao’, while it was 32.0% in the seed oil of ‘Liantai’. Thus, it is difficult to distinguish between the two peony seed oils according to the content of α-linolenic acid, which might be a quantitative characteristic of *Paeonia* seed oils.

### 3.2. Sterol Profiles and Other Nonpolar Compounds in Paeonia Seed Oils

A total of 13 compounds, including 11 phytosterols, squalene and γ-tocopherol, were detected in all samples, as shown in Table 2. The total sterol content varied among tree peony seed oils, between 2970.65 and 4817.23 mg/kg oil, with *P. rockii* from Gansu DingXi having the highest, while *P. ostii* from Jiangsu Shuyang had the lowest, which is at the middle level compared with that of all the named vegetable oils in the Codex Alimentarius (CXS 210–1999). β-sitosterol (1803–2794 mg/kg) and fucosterol (682–1225 mg/kg) were the major phytosterols in all the tree peony seed oils tested, although there was large continuous variation in the contents. In addition, the total contents of phytosterols in ‘Tuopanxianbao’ and ‘Liantai’ seed oils were in the variation range of the tree peony seed oil. Generally, the 11 phytosterols showed continuous variation in the tree and herbaceous peony seed oils, especially β-sitosterol and fucosterol, which might be considered as part of the compositional index for *Paeonia* seed oils, since there is rarely a similar distribution reported in other vegetable oils, to the best of our knowledge.

Similar to the total content of phytosterols, the content of γ-tocopherol showed continuous variation among all the tree and herbaceous peony seed oils tested. As for the tree peony seed oil, the highest content of γ-tocopherol (55.18 mg/kg) was from *P. rockii* in Gansu DingXi, while the lowest (22.07 mg/kg) was from *P. ostii* in Jiangsu Tongling. Squalene ranged from 26.58 mg/kg (*P. ostii* in Shandong Heze) to 55.72 mg/kg (*P. ostii* in Hubei Wuhan). Notably, the content of squalene was much higher in herbaceous peony seed oil, i.e., 115.21 mg/kg in ‘TuoPanXianBao’ and 111.84 mg/kg in ‘LianTai’.

### 3.3. Phenolic Compounds in Paeonia Seed Oils

#### 3.3.1. Qualitative Analysis

**Figure 2:**
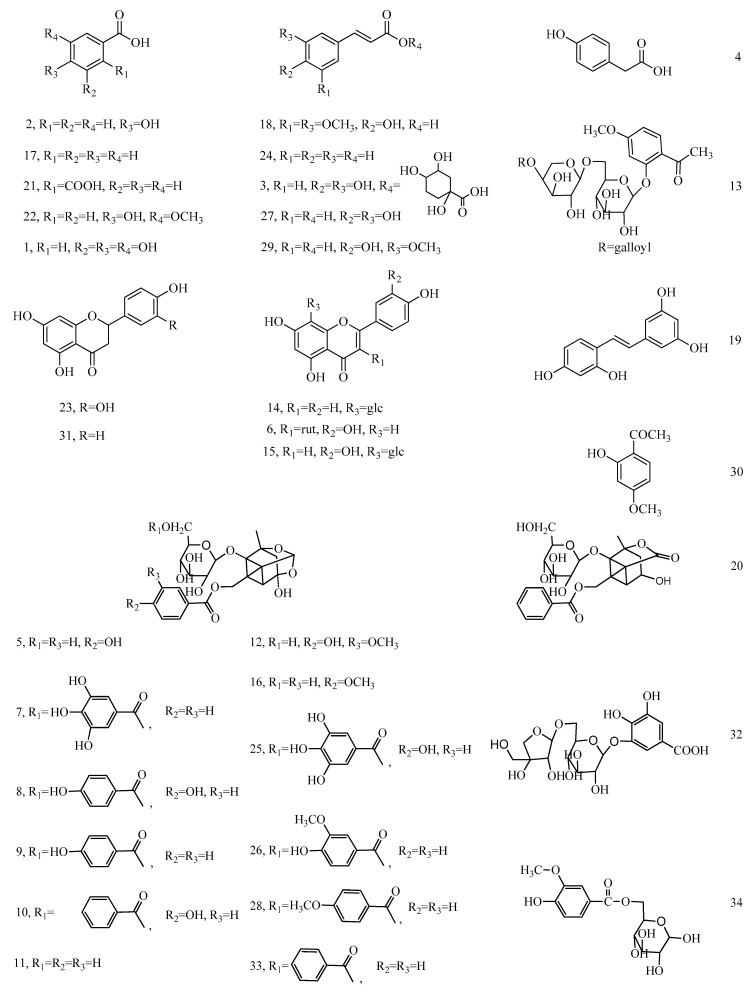
Chemical structures of 34 compounds in *Paeonia* seed oil identified by UHPLC-Q-TOF-MS. Glc, glucosyl; Rut, rutinose. The numbers of the compounds correspond to the peak numbers in Table 3.

UHPLC-Q-TOF-MS and -MS/MS was applied to characterize the phenolic composition in seed oils, and it was based on mass accurate data, which provide the molecular formula of the compounds. Their mass fragmentation patterns were also studied in depth to finally establish their structures, which are outlined below. Totally, 34 phenolic compounds were tentatively identified in the extracts of the *Paeonia* seed oils, as Table 3 and Figure 2 shows.

*Phenolic acids*. The identification of phenolic acids was as reported in our previous study [11]. The following classes were identified in *Paeonia* seed oils: benzoic acids (5), cinnamic acids (5) and a *p*-hydroxyphenylacetic acid. Generally, the phenolic acids share the same fragmentation characteristics in the negative mode under the ESI source, i.e., they lost -CO_2_ and gained fragment ions [M-H-44]- in the MS/MS spectra [11]. Some of these phenolic acids have previously been reported in *Paeonia* plants, agreeing with our results. As an example, benzoic, gallic and caffeic acids were reported in the seeds of all the nine tree peony species native to China [15]. Paeonol and benzoic acid were found to be the active constituents (with acaricidal activity) of *Paeonia suffruticosa* root bark by spectroscopic analyses [16].

*Flavonoids*. Five flavonoids were identified in *Paeonia* seed oils, including vitexin, rutin, orientin, eriodictyol and naringenin. Peak 6 (*m/z* 609.1446) generated two fragment ions, at *m/z* 300.0011 and 301.0121 (quercetin), after the precursor [M-H]^−^ lost the disaccharide moiety, -C_12_H_20_O_9_ and -C_12_H_21_O_9_, respectively. This compound was therefore characterized as rutin, with the typical fragmentation pattern of the flavonoids *O*-glycosides [17]. Alternatively, the ion at *m/z* 431.0960 (peak 14) reported a molecular formulae C_21_H_20_O_10_. The parent ion [M-H]^−^ fragmented on the glucose moiety, and got the ion [M-H-C_3_H_6_O_3_]^−^ at *m/z* 341.2250. Then, it lost CH-OH (30 Da) twice, [M-H-C_4_H_8_O_4_]^−^ at *m/z* 311.1516 and [M-H-C_5_H_10_O_5_]^−^ at *m/z* 281.1583, which was reported in the identification of vitexin [18,19]. Similarly, peak 15 at *m/z* 431.0960 gave a molecular formulae C_21_H_20_O_11_, the compound orientin possibly being inferred, with reference to our in-house database [20]. Three fragment ions, *m/z* 357.2028, 327.2799 and 297.0389, were obtained after the precursor [M-H]- lost -C_3_H_6_O_3_, -C_4_H_8_O_4_ and -C_5_H_10_O_5_, respectively, during the 0,3, 0,2 and 0,1 bond break of the glycoside ring during MS/MS analysis. On the basis of its *m/z* and molecular formulae, peak 31 (*m/z* 271.0614, C_15_H_12_O_5_) was characterized tentatively as naringenin, and its fragmentation process was reported widely as a common flavonoid, with fragmentation process IV. Similarly, the compound of the peak 23 (*m/z* 287.0560) was identified as eriodictyol [20]. The basic fragmentation process was almost the same. The difference was that the fragment ion *m/z* 151.0033 continued to lose -CO_2_, and got the third fragment *m/z* 107.0133 for naringenin. Among the flavonoids above, rutin was reported in the seeds of these two tree peony species [15].

*Monoterpene glycosides*. The structural characteristics included the possession of the “cage-like” pinane skeleton, with different substituent groups, typically glucosyl and phenyl-containing groups [21]. In addition, one feature of the fragmentation in MS/MS was distinctive for the monoterpene glycoside. That is, the fragments at *m/z* 121, *m/z* 137, *m/z* 151 and *m/z* 169 corresponded to benzoic acid, *p*-hydroxybenzoic acid, *p*-methoxybenzoic acid and gallic acid, respectively, which are the substituents on pinane of the aglycones together with hexose, in the case of glycosides, as Table 3 shows. Take paeoniflorin (peak 11) as an example; [M + HCOO]^−^ was detected at *m/z* 525.1617 and supplied the possible molecular formula C_24_H_29_O_13_, as indicated by the software. On the basis of the in-house database, we may infer that it was the adduct of paeoniflorin or albiflorin with formic acid (from the mobile phase). Thus, the compound was inferred as possibly being paeoniflorin or albiflorin, since these share the same molecular formulae, which corresponds to C_23_H_28_O_11_. Its corresponding ion was also detected at *m/z* 479.1558. Through being compared with a previous report about paeoniflorin [22], the fragments shown in Table 3 were consistent with the MS/MS fragmentation of paeoniflorin. In this sense, *m/z* 479.1559 was the molecular ion peak of [M-H]^−^ of paeoniflorin, while *m/z* 449.1927 was the rearrangement ion after the pinane skeleton lost a -CH_2_O. Then, it continued losing a benzoic acid and the fragment *m/z* 327.1803 was obtained. In another case, a fragment at *m/z* 357.1182 was formed by the molecular ion peak losing benzoic acid; then, this fragment ion continued losing the glucose moiety (162 u) (*m/z* 195.0655). Finally, a further loss of -CH_2_O on the pinane skeleton produced the rearrangement ion at *m/z* 165.0875. The benzoic acid was also detected at *m/z* 121.0527, as commented before, suggesting the presence of this substituent. All the fragments suggested that this compound was paeoniflorin, finally confirmed with the commercial standard. Subsequently, the compound at the peak 20(R_t_ 5.357 min), with the same molecular formula, was tentatively identified as albiflorin. The fragments at *m/z* 435.1389 and 327.0819 were obtained from the precursor ion at *m/z* 479.1559 losing -CO_2_ and a benzoic acid, respectively. Then, the benzoic acid combined with the glucose moiety (162 u), and got the rearrangement ion at *m/z* 283.1088. The fragmentation was the same as that previously reported for albiflorin [23].

Another example was oxypaeoniflorin (peak 5). The compound (R_t_ 3.223 min), with the molecular formula C_23_H_28_O_12_ the precursor ion at *m/z* 495.1508 in the ESI mode, was tentatively proposed as oxypaeoniflorin [23]. In the MS/MS spectrum, this compound produced a fragment ion [M-H-pOHBA] at *m/z* 357.0649, after the parent ion lost *p*-hydroxybenzoic acid; at the same time, the p-hydroxybenzoic acid fragment [pOHBA-H] was detected at *m/z* 137.0494. On the other hand, the molecular ion peak lost the glucose moiety (162 u) and obtained the fragment ion [M-H-Glc] at *m/z* 333.0500. Similarly, the compounds at peaks 7, 10, 25 and 33 were identified as galloylpaeoniflorin, benzoyloxypaeoniflorin, galloyloxypaeoniflorin, and benzoylpaeoniflorin, respectively, having lost the different substitute groups [22,23,24]. Another group of compounds, such as mudanpioside A, C, D, E, H and J, were characterized according to the MS and MS/MS information of peaks 28, 9, 16, 12, 8 and 26 and similar fragmentation characteristics, for example, losing -CH_2_O, the benzoyl group and *p*-methoxy benzoic acid [22].

*Paeonol derivatives*. Paeonol and three derivatives have been detected in *Paeonia* seed oils. The compound at peak 30 (R_t_ 7.58 min), with a parent ion at *m/z* 165.0558, had fragment ions at *m/z* 150.0598 and 135.1075, which were obtained by consecutively losing -CH_3_. Then, a -CH was lost on the skeleton of phenolic glycoside, and the fragment ion at *m/z* 122.0603 was produced. Based on the information above, the compound was proposed as paeonol, and its fragmentation process has been reported once before [22]. Similarly, the compounds at the peak 32 (*m/z* 463.1814) and peak 34 (*m/z* 329.1248) reported their fragment ions by consecutively losing -C_2_H_4_O_2_ and -CH_3_ twice, respectively. For the compound at peak 32, the product ions at *m/z* 403.3115 and 343.5776 were obtained. For peak 34, the two fragment ions were reported at *m/z* 314.3025 and 299.1015. Then, the fragment ion at *m/z* 271.2795 was produced by losing -CO from the fragment at *m/z* 299.1015. Thus, the compounds at peaks 32 and 34 were identified as mudanoside A and B, respectively (Xu, et al., 2006). The compound at peak 13 (R_t_ 4.77 min), with *m/z* 611.1614, was tentatively identified as either suffruticoside A or C, based on the report by Yoshikawa and co-workers [24].

*Stilbenoids*. Peak 19 (R_t_ 5.27 min) at *m/z* 243.0667 was assumed to be C_14_H_12_O_4_; probably the molecular formulae of oxyresveratrol, with reference to the in-house database. The fragmentation process was as follows: the precursor [M-H]^−^ lost -H_2_O and got the fragment *m/z* 225.1162 ([M-H-H_2_O]^−^); then, the fragment continued losing -C_2_H_2_ (26 Da), and produced the fragment *m/z* 199.1161 ([M-H-H_2_O-C_2_H_2_]^−^). In addition, the precursor [M-H]^−^ lost -C_3_H_4_O_2_, and got the fragment *m/z* 175.0011 ([M-H-C_3_H_4_O_2_]^−^); this fragment continued losing H_2_O and produced a fragment ion at *m/z* 157.0022. All the fragmentations were consistent with the report about oxyresveratrol [25].

It is notable to highlight that all the 34 phenolic compounds were reported for the first time in tree and herbaceous peony seed oils. The monoterpene glycosides and paeonol derivatives, called “*Paeonia* glycosides”, as a group reported widely as the main bioactive components in Moutan Cortex and other herbal medicines from *Paeonia* plants [21], have not been reported in other vegetable oils yet.

#### 3.3.2. Quantitative Analysis

The semi-quantitative results obtained by UHPLC-Q-TOF-MS/MS were expressed as micrograms per gram of oil. Globally, 34 phenolic compounds were all semi-quantified according to the chemical standards that share similar chemical structures [15].

**Figure 3:**
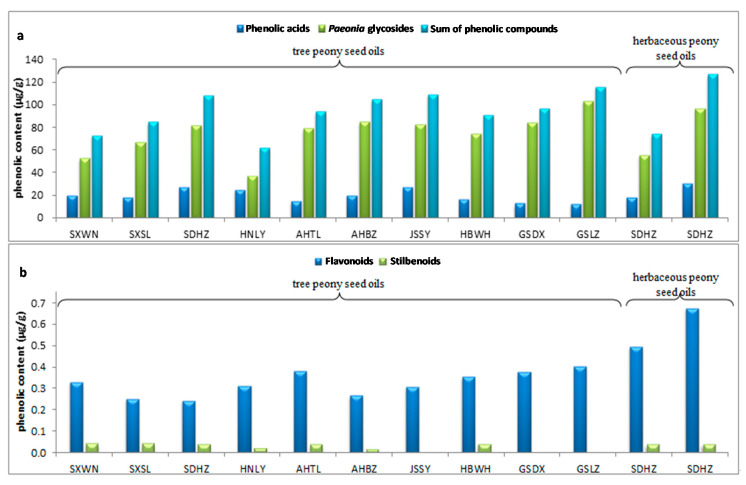
(**a**) Content of phenolic acids, *Paeonia* glycosides and the sum of phenolic compounds, as well as (**b**) content of flavonoids and stibenoids in tree and herbaceous peony seed oils from different sampling sites. In herbaceous peony, SDHZ refers to samples ‘Tuopanxianbao’ and ‘Liantai’, respectively.

The benzoic acids (benzoic acid, *p*-hydroxybenzoic acid, phthalic acid, vanillic acid and gallic acid) were quantified with benzoic acid; cinnamic acids (cinnamic acid, caffeic acid, ferulic acid, sinapic acid and chlorogenic acid) were quantified with cinnamic acid; *p*-hydroxyphenylacetic acid was quantified absolutely with the corresponding standard; all the flavonoids, including naringenin, eriodictyol, vitexin, orientin and rutin, were quantified with naringenin; paeoniflorin was used to quantify all the *Paeonia* glycosides; paeonol and oxyresveratrol were quantified absolutely with the corresponding standards. This quantitative strategy is generally done in the literature, due to the unavailability of phenolic standards in most of the cases. Table 4 shows the individual compositions and Figure 3 shows the total contents of phenolic compounds of different classes, highlighting that the main classes were phenolic acids and *Paeonia* glycosides.

*Phenolic acids*. The content of 11 phenolic acids ranged between 12.29 and 26.71 μg/g in tree peony seed oils; the content in *P. rockii* seed oils was lower than in *P. ostii* seed oils. The main phenolic acid was benzoic acid, whose concentration varied between 3.52 and 7.59 μg/g. Another main compound, sinapic acid, ranged from 1.75 to 5.25 μg/g. The content of benzoic acid was reported to be up to 15.31 mg/100 g dry seeds of *P. rockii* and 15.98 mg/100 g dry seeds of *P. ostii*. Gallic acid and caffeic acid were also present in *P. rockii* and *P. ostii* seeds at lower concentration [15]. Obviously, the seeds contain phenolic acids, and these compounds are transferred during oil extraction.

*Flavonoids*. The total content of five flavonoids ranged from 0.24 to 0.67 μg/g, and all of them could be treated as trace compounds, but each compound was detected in all the samples investigated. Among them, rutin (0.03–0.27 μg/g herein) was also detected in the seeds of two subspecies of *P. rockii* (33.65 and 26.2 mg/100g dry seeds) and *P. ostii* (3.86 mg/100g dry seeds) [15].

*Monoterpene glycosides and paeonol derivatives: “Paeonia glycosides”*. These compounds in tree peony seed oils were composed of 13 monoterpene glycosides and 4 paeonol derivatives, which occurred ubiquitously in all the samples investigated. Most of them were rarely found in other plants, and were regarded as the characteristic chemotaxonomic marker of *Paeonia* plants [26]. The total content ranged from 36.62 μg/g (*P. ostii* from Henan Luoyang) to 103.17 μg/g (*P. rockii* from Gansu Lanzhou), with 81.9–95.4% monoterpene glycosides. Among them, the major compound was paeoniflorin; its content ranging from 23.05 μg/g (*P. ostii*) to 86.55 μg/g (*P. rocki*), which accounts for 67.3–88.0% of the content of monoterpene glycosides. It was notable that the paeoniflorin in the seed oils of *P. rockii* (69.27 μg/g and 86.55 μg/g) was higher than that in *P. ostii*. Meanwhile, paeoniflorin varied from 23.04 to 64.62 μg/g in the seed oils of *P. ostii*. Paeoniflorin was also reported in the seeds of *P. rockii* and *P. ostii*, which were in higher quantities than the aforementioned phenolic compounds (15.03 mg/g dry and 15.57 mg/g dry seeds [27], respectively) as we also observed in the oils. Albiflorin, paeoniflorin and suffruticosol A and C were also found in the seed coat and kernel of *P. ostii*, which explains their presence in the oil [28]. Moreover, as for phenolic glycosides, mudanoside A (1.55–7.35 μg/g) and paeonol (1.30–5.84 μg/g) were the main compounds.

*Stilbenoids*. The content of oxyresveratrol ranged from 0.01 to 0.04 μg/g in the tree peony seed oil.

Regarding herbaceous peony seed oils, their phenolic acids were 17.74 and 29.96 μg/g, while the flavonoids were also very low; 0.50 and 0.67 μg/g. As for the characteristic compounds of *Paeonia* plants, the content of monoterpene glycosides was 50.18 and 90.27 μg/g, and the content of paeonol derivatives was 5.37 and 6.17 μg/g, which were all in the range found in *P. rockii* and *P. ostii* seed oils. Generally, the phenolic composition in *Paeonia* seed oils was basically uniform, and the “*Paeonia* glycosides” were the main constituents, especially paeoniflorin.

Clearly, the total contents of the phenolic compounds in *Paeonia* seed oils are shown in Figure 3a,b, which are comparable with those of extra virgin olive oil, camellia seed oil and tea seed oil, although the phenolic composition is different compared to these vegetable oils [11,29].

Notably, these glycosides are the major active components in the Chinese Medicinal Materials from the root bark of tree peony (Moutan Cortax) and the root of herbaceous peony (Radix Paeonia Alba or Rubra). Thus, the tree and herbaceous peony seed oils might have corresponding antioxidative [24], anti-inflammation [30], antitumor [31], antidepressant [32] and antithrombotic [33] activities reported in these herbal medicines with the same active components. Paeoniflorin was reported in vegetable oils for the first time herein, and its content was 23.05–86.55 μg/g, which was higher than many other phenolic compounds reported in vegetable oils, to the best of our knowledge. Thus, paeoniflorin might be regarded as the representative of “*Paeonia* glycosides”, which is the most important molecular marker of compositional characteristics. Although some studies have attributed the functional properties of peony seed oil to its content of unsaturated fatty acids [1,34], future studies should also consider the latter minor components, which have not been characterized until now. Moreover, due to their phenolic nature, these compounds can contribute not only to the functional properties of the peony seed oils, but also to their oxidative stability, and therefore more work is required. Further studies could also consider oil from seeds of other peony species, such as *Paeonia anomala* subsp. *veitchii* (Lynch) D.Y.Hong and K.Y.Pan (or *Paeonia veitchii* Lynch), which also presents “*Paeonia* glycosides” [35].

### 3.4. HCA of Paeonia Seed Oils Based on Their Composition

**Figure 4:**
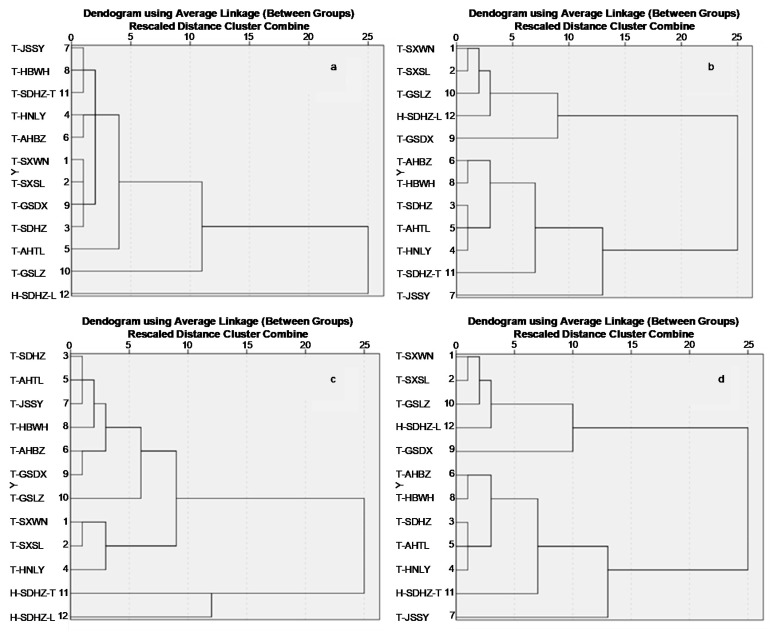
Hierarchical cluster analysis of *Paeonia* seed oils based on the data of composition: (**a**) fatty acids; (**b**) squalene, γ-tocopherol and sterols; (**c**) phenolic compounds; (**d**) all the components.

To distinguish between tree and herbaceous peony seed oils, HCA was performed on the basis of the fatty acids, sterols and phenolic compounds, respectively (Figure 4a–c). The results showed both of them clustered together, except for in the HCA on the phenolic compounds. However, it is unfeasible to make a distinction between tree and herbaceous peony seed oils through qualifying and quantifying the phenolic compounds via UHPLC-MS in practice. Finally, combining all the components, HCA shows that all the tree peony seed oils were clustered together with the herbaceous peony seed oils in the dendrogram (Figure 4d). Although *P. rockii* seed oil had higher levels of α-linolenic acid, β-sitosterol, fucosterol and paeoniflorin, the two samples were still grouped with other tree peony seed oils. Notably, the herbaceous peony seed oils branched from each other, but clustered with tree peony seed oils. HCA summarized the compositional analysis of *Paeonia* seed oils, and showed continuous variation in the composition, but the work supplied the theoretical reliability of protected designation of origin (PDO) for peony seed oils.

## 4. Conclusions

A total of 17 fatty acids, 13 sterols, including squalene and γ-tocopherol, and 34 phenolic compounds found in tree and herbaceous peony seed oils have been characterized in this work. Clearly, the tree peony seed oil was rich in active compounds, for instance, α-linolenic acid (39.0–48.3%), β-sitosterol (1802.5–2793.7 mg/kg) and especially paeoniflorin (23.05–86.55 μg/g), which differentiates these oils from other vegetable oils except for herbaceous peony seed oils. Moreover, this work collected the data regarding fatty acids, phytosterols and phenolic compounds in tree peony seed oils from all the main cultivation areas in China, which outlined the compositional characteristics of tree peony seed oil. Comparatively, it is difficult to distinguish between tree and herbaceous peony seed oils due to their continuous variation in composition. Peony seed oil is suggested for *Paeonia* seed oil as an official English name, and it is a distinctive new resource of oil with paeonol and “*Paeonia* glycosides” as molecular markers, which highlight potential functions related to the homology between medicine and food.

## Figures and Tables

**Table 1:** Relative fatty acid composition (%) of 10 *Paeonia* seed oil samples ^1^.

Cultivar/Species	*P. ostii*								*P. rockii*		TuoPanXianBao	LianTai
Region	SXWN	SXSL	SDHZ	HNLY	AHTL	AHBZ	JSSY	HBWH	GSDX	GSLZ	SDHZ	SDHZ
C16:0	5.80 ± 0.00 ^c^	5.88 ± 0.00 ^a^	5.53 ± 0.00 ^h^	5.34 ± 0.00 ^j^	5.73 ± 0.00 ^d^	5.55 ± 0.00 ^g^	5.70 ± 0.00 ^e^	5.70 ± 0.00 ^f^	5.82 ± 0.00 ^b^	4.94 ± 0.00 ^k^	5.50 ± 0.00 ^i^	3.88 ± 0.00 ^l^
C18:0	1.97 ± 0.00 ^b^	2.26 ± 0.00 ^a^	1.74 ± 0.00 ^i^	1.78 ± 0.00 ^g^	1.81 ± 0.00 ^f^	1.75 ± 0.00 ^h^	1.86 ± 0.00 ^d^	1.85 ± 0.00 ^e^	1.87 ± 0.00 ^c^	1.65 ± 0.00 ^j^	1.87 ± 0.00 ^c^	0.81 ± 0.00 ^k^
C18:1n9	21.76 ± 0.01 ^h^	21.44 ± 0.00 ^i^	21.89 ± 0.01 ^g^	20.63 ± 0.01 ^l^	22.09 ± 0.01 ^f^	21.01 ± 0.01 ^k^	23.81 ± 0.00 ^d^	23.79 ± 0.00 ^e^	21.34 ± 0.00 ^j^	26.78 ± 0.00 ^b^	23.83 ± 0.01 ^c^	31.35 ± 0.01 ^a^
C18:2n6	24.60 ± 0.00 ^h^	23.98 ± 0.00 ^i^	25.72 ± 0.00 ^e^	27.754 ± 0.01 ^c^	29.94 ± 0.00 ^b^	27.67 ± 0.00 ^d^	25.02 ± 0.00 ^g^	25.04 ± 0.02 ^f^	23.25 ± 0.01 ^k^	16.91 ± 0.00 ^l^	23.62 ± 0.00 ^j^	30.74 ± 0.01 ^a^
α-C18:3n3	44.38 ± 0.02 ^d^	44.94 ± 0.01 ^c^	43.59 ± 0.04 ^f^	43.04 ± 0.02 ^g^	38.97 ± 0.01 ^k^	42.54 ± 0.01 ^h^	42.11 ± 0.00 ^j^	42.15 ± 0.02 ^i^	46.24 ± 0.01 ^b^	48.31 ± 0.00 ^a^	43.73 ± 0.00 ^e^	31.97 ± 0.01 ^l^
SFA	8.17	8.56	7.64	7.51	7.90	7.67	7.91	7.89	8.09	6.98	7.72	4.89
MUFA	22.37	22.01	22.51	21.23	22.68	21.60	24.61	24.40	21.94	27.39	24.43	32.48
PUFA	69.46	69.43	69.87	71.35	69.42	70.73	67.65	67.70	69.97	65.63	67.86	62.63
UFA	91.83	91.44	92.38	92.57	92.10	92.33	92.27	92.11	91.91	93.02	92.28	95.11

^1^ Values are means ± standard deviations, *n* = 3. Different superscript letters within the same row indicate significant differences (one-way ANOVA and Duncan test, *p* ≤ 0.05). SFA, saturated fatty acids; MUFA, monounsaturated fatty acids; PUFA, polyunsaturated fatty acids; UFA, unsaturated fatty acids.

**Table 2:** Squalene, γ-tocopherol and phytosterol contents (mg/kg) of 10 *Paeonia* seed oil samples ^1^.

Cultivar/Species	*P. ostii*								*P. rockii*		TuoPanXianBao	LianTai
Region	SXWN	SXSL	SDHZ	HNLY	AHTL	AHBZ	JSSY	HBWH	GSDX	GSLZ	SDHZ	SDHZ
Squalene	30.84 ± 0.59 ^e^	46.65 ± 0.45 ^c^	26.58 ± 1.50 ^f^	40.35 ± 0.57 ^d^	31.20 ± 1.00 ^e^	45.90 ± 0.91 ^c^	43.22 ± 2.30 ^cd^	55.72 ± 2.41 ^b^	39.95 ± 0.80 ^d^	41.05 ± 2.46 ^d^	115.21 ± 5.89 ^a^	111.84 ± 3.45 ^a^
γ-tocopherol	32.95 ± 0.62 ^f^	37.44 ± 2.07 ^e^	28.27 ± 0.26 ^g^	32.28 ± 1.28 ^f^	22.07 ± 0.60 ^h^	47.38 ± 1.70 ^c^	31.49 ± 1.19 ^f^	24.81 ± 1.03 ^h^	55.18 ± 1.17 ^b^	39.54 ± 3.09 ^e^	43.29 ± 1.08 ^d^	59.32 ± 0.67 ^a^
Cholesterol	25.74 ± 1.71 ^b^	20.35 ± 1.16 ^de^	17.23 ± 0.08 ^fg^	16.72 ± 1.49 ^fg^	22.07 ± 0.52 ^cd^	24.49 ± 0.33 ^bc^	22.69 ± 2.33 ^cd^	22.31 ± 2.97 ^cd^	30.95 ± 0.44 ^a^	14.80 ± 0.02 ^g^	18.71 ± 0.19 ^ef^	32.70 ± 1.63 ^a^
Campesterol	51.60 ± 1.88 ^e^	59.47 ± 0.70 ^d^	51.72 ± 1.11 ^e^	37.70 ± 1.32 ^h^	51.77 ± 0.39 ^e^	41.36 ± 4.57 ^gh^	44.92 ± 1.74 ^fg^	49.21 ± 2.12 ^ef^	72.81 ± 0.60 ^c^	115.03 ± 4.29 ^b^	155.16 ± 4.91 ^a^	70.56 ± 3.72 ^c^
Pregnanediol	20.45 ± 1.10 ^c^	20.68 ± 0.29 ^c^	6.33 ± 0.72 ^f^	15.70 ± 0.58 ^e^	17.21 ± 4.46 ^de^	17.19 ± 2.16 ^de^	20.14 ± 0.49 ^cd^	21.20 ± 1.37 ^bc^	28.30 ± 1.48 ^a^	7.24 ± 0.67 ^f^	19.08 ± 0.79 ^cd^	23.97 ± 1.50 ^b^
β-sitosterol	2298 ± 9 ^cd^	2368 ± 42 ^c^	2274 ± 41 ^d^	2018 ± 2 ^f^	2137 ± 21 ^e^	2010 ± 47 ^f^	1803 ± 55 ^g^	2091 ± 6 ^ef^	2794 ± 61 ^a^	2330 ± 44 ^cd^	2043 ± 18 ^f^	2482 ± 46 ^b^
Fucosterol	1123 ± 25 ^b^	1098 ± 24 ^b^	965 ± 40 ^d^	990 ± 4 ^d^	1036 ± 11 ^c^	854 ± 39 ^f^	682 ± 23 ^h^	766 ± 18 ^g^	1225 ± 34 ^a^	1198 ± 22 ^a^	566 ± 9 ^i^	903 ± 4 ^e^
∆^5^-Avenasterol	22.90 ± 0.50 ^c^	18.95 ± 1.18 ^d^	17.95 ± 1.97 ^de^	17.60 ± 0.34 ^de^	18.58 ± 1.02 ^d^	31.64 ± 1.23 ^a^	16.21 ± 2.14 ^e^	19.13 ± 0.30 ^d^	26.26 ± 0.54 ^b^	26.42 ± 0.54 ^b^	10.00 ± 0.10 ^f^	18.34 ± 1.00 ^d^
∆^7^-Avenasterol	51.56 ± 2.78 ^f^	77.88 ± 2.48 ^d^	35.51 ± 0.79 ^h^	66.75 ± 0.95 ^e^	23.04 ± 2.37 ^i^	53.70 ± 1.92 ^f^	35.28 ± 1.55 ^h^	37.42 ± 1.02 ^gh^	41.67 ± 0.77 ^g^	164.54 ± 7.01 ^b^	275.12 ± 5.01 ^a^	107.69 ± 2.73 ^c^
Obtusifoldienol	88.48 ± 1.95 ^ab^	88.64 ± 2.15 ^ab^	41.72 ± 0.59 ^f^	68.66 ± 0.47 ^d^	67.16 ± 3.44 ^d^	92.16 ± 7.45 ^a^	40.98 ± 3.06 ^f^	49.46 ± 4.73 ^e^	83.30 ± 1.06 ^b^	76.56 ± 0.68 ^c^	21.53 ± 0.18 ^h^	32.41 ± 0.87 ^g^
Cycloartenol	337.88 ± 18.45 ^a^	326.36 ± 2.86 ^a^	200.18 ± 1.89 ^cd^	203.94 ± 1.25 ^c^	197.48 ± 3.14 ^cde^	228.12 ± 12.78 ^b^	161.67 ± 5.67 ^f^	184.43 ± 11.28 ^e^	228.44 ± 5.61 ^b^	188.62 ± 0.48 ^de^	58.58 ± 0.64 ^g^	229.46 ± 5.72 ^b^
Betulin	185.96 ± 5.64 ^a^	121.99 ± 1.13 ^bc^	123.19 ± 1.86 ^bc^	134.08 ± 5.72 ^b^	132.41 ± 0.38 ^b^	121.98 ± 5.35 ^bc^	75.89 ± 2.88 ^de^	81.90 ± 3.25 ^d^	122.34 ± 2.03 ^bc^	64.02 ± 2.09 ^e^	190.21 ± 1.22 ^a^	112.71 ± 3.89 ^c^
Lanosterol	128.61 ± 9.22 ^e^	184.85 ± 1.10 ^b^	85.76 ± 2.13 ^f^	136.44 ± 4.91 ^e^	149.33 ± 0.29 ^d^	84.37 ± 5.25 ^f^	68.10 ± 0.64 ^g^	134.84 ± 4.78 ^e^	164.41 ± 3.00 ^c^	248.53 ± 7.89 ^a^	62.71 ± 4.22 ^g^	188.13 ± 3.63 ^b^
Total phytosterol	4334.60	4385.12	3818.87	3706.54	3852.00	3560.33	2970.65	3457.07	4817.23	4433.52	3420.26	4200.56

^1^ Values are means ± standard deviations, *n* = 3. Different superscript letters within the same row indicate significant differences (one-way ANOVA and Duncan test, *p* ≤ 0.05).

**Table 3:** Summary of phenolic compounds characterized in *Paeonia* seed oil samples using UHPLC-Q-TOF-MS/MS.

Peak	R_t_ (min)	Formula	Exp. *m/z*	Calc. *m/z*	Error (ppm)	MS/MS Ions	Compound	Reference
1	0.71	C_7_H_6_O_5_	169.0147	169.0142	2.96	125.0339	Gallic acid	[15]
2	2.59	C_7_H_6_O_3_	137.0246	137.0244	1.46	93.0336	*p*-Hydroxybenzoic acid	[15]
3	2.93	C_16_H_18_O_9_	353.0874	353.0878	−1.13	191.0548	Chlorogenic acid	[15]
4	3.13	C_8_H_8_O_3_	151.0403	151.0401	1.32	107.0497	*p*-Hydroxy phenylacetic acid	[15]
5	3.22	C_23_H_28_O_12_	495.1508	495.1508	0.00	357.0649, 137.0494, 333.0500	Oxypaeoniflorin	[23]
6	3.26	C_27_H_30_O_16_	609.1446	609.1456	−1.64	300.0011, 301.0121	Rutin	[15]
7	3.26	C_30_H_32_O_15_	631.1658	631.1668	−1.58	613.4873, 491.3507, 169.1158, 125.1254	Galloylpaeoniflorin	[24]
8	4.00	C_30_H_32_O_14_	615.1739	615.1719	3.25	585.4402, 477.3758, 447.2843, 137.0231	Mudanpioside H	[22]
9	4.37	C_30_H_32_O_13_	599.1790	599.1770	3.34	477.2317, 121.2042	Mudanpioside C	[22]
10	4.38	C_30_H_32_O_13_	599.1961	599.1964	−0.50	477.1955, 449.2358, 315.1011	Benzoyloxy paeoniflorin	[22]
11	4.48	C_23_H_28_O_11_	479.1558	479.1559	−0.21	449.1927, 357.1182, 327.1803, 195.0655, 165.0875, 121.0527	Paeoniflorin	[22]
12	4.50	C_24_H_30_O_13_	525.1617	525.1614	0.57	495.1454, 357.1171	Mudanpioside E	[22]
13	4.77	C_27_H_32_O_16_	611.1614	611.1618	−0.65	462.0420, 169.1193, 151.0693, 125.1209	Suffruticoside A or C	[24]
14	4.83	C_21_H_20_O_10_	431.0960	431.0984	−5.57	341.2250, 311.1516, 281.1583	Vitexin	[19]
15	4.968	C_21_H_20_O_11_	447.0932	447.0933	−0.22	357.2028, 327.2799, 297.0389	Orientin	[20]
16	5.06	C_24_H_30_O_12_	509.1667	509.1664	0.59	357.2204, 327.2506, 151.2981	Mudanpioside D	[22]
17	5.14	C_7_H_6_O_2_	121.0295	121.0295	0.00	77.0400	Benzoic acid	Standard
18	5.20	C_11_H_12_O_5_	223.0614	223.0612	0.90	208.0384, 163.0401	Sinapic acid	[15]
19	5.27	C_14_H_12_O_4_	243.0667	243.0663	1.65	225.1162, 199.1161, 175.0011, 157.0022	Oxyresveratrol	[25]
20	5.36	C_23_H_28_O_11_	479.1559	479.1559	0.00	435.1389, 327.0819, 283.1088	Albiflorin	[23]
21	5.90	C_8_H_6_O_4_	165.0193	165.0193	0.00	121.0293, 77.0393	Phthalic acid	[11]
22	6.05	C_8_H_8_O_4_	167.0350	167.0350	0.00	152.0113, 123.0458, 108.0210	Vanillic acid	[15]
23	6.07	C_15_H_12_O_6_	287.0560	287.0561	−0.35	151.0024, 135.0435	Eriodictyol	[20]
24	6.21	C_9_H_8_O_2_	147.0450	147.0452	−1.36	103.0545	Cinnamic acid	[15]
25	6.33	C_30_H_32_O_16_	647.1692	647.1618	−0.93	509.2651, 449.5288	Galloyloxypaeoniflorin	[22]
26	6.43	C_31_H_34_O_14_	629.1874	629.1876	−0.32	599.2841, 507.1883	Mudanpioside J	[22]
27	7.15	C_9_H_8_O_4_	179.0348	179.0350	−1.12	135.0454	Caffeic acid	[15]
28	7.19	C_31_H_34_O_13_	613.1922	613.1927	−0.82	583.2593, 431.2965	Mudanpioside A	[22]
29	7.47	C_10_H_10_O_4_	193.0506	193.0506	0.00	178.0262, 149.0602, 134.0376	Ferulic acid	[15]
30	7.58	C_9_H_10_O_3_	165.0558	165.0557	0.61	150.0598, 135.1075, 122.0603	Paeonol	[22]
31	7.99	C_15_H_12_O_5_	271.0614	271.0612	0.74	151.0033, 119.0503, 107.0133	Naringenin	Standard
32	8.03	C_18_H_24_O_14_	463.1814	463.1821	−1.51	403.3115, 343.5776	Mudanoside B	[23]
33	9.29	C_30_H_32_O_12_	583.1827	583.1821	1.03	553.3238, 535.4236	Benzoylpaeoniflorin	[23]
34	9.43	C_15_H_22_O_8_	329.1248	329.1242	1.82	314.3025, 299.1015, 271.2795	Mudanoside A	[23]

R_t_, retention time; Exp. *m/z*, experimental *m/z* for [M − H]^−^; Calc. *m/z*, calculated *m/z* for [M −H]^−^.

**Table 4:** Phenolic composition (μg/g) of 10 *Paeonia* seed oil samples from different areas ^1^.

Cultivar/Species	*P. ostii*								*P. rockii*		Tuopanxianbao	Liantai
Region	SXWN	SXSL	SDHZ	HNLY	AHTL	AHBZ	JSSY	HBWH	GSDX	GSLZ	SDHZ	SDHZ
Benzoic acid	3.665 ± 0.012 ^j^	3.519 ± 0.008 ^k^	7.591 ± 0.053 ^a^	5.845 ± 0.077 ^e^	6.417 ± 0.008 ^c^	5.411 ± 0.005 ^f^	7.322 ± 0.035 ^b^	6.223 ± 0.104 ^d^	4.222 ± 0.022 ^h^	4.577 ± 0.012 ^g^	3.589 ± 0.016 ^k^	4.024 ± 0.025 ^i^
*p*-Hydroxybenzoic acid	1.257 ± 0.004 ^f^	1.243 ± 0.007 ^f^	2.566 ± 0.005 ^c^	2.667 ± 0.009 ^b^	1.470 ± 0.001 ^d^	3.041 ± 0.008 ^a^	2.679 ± 0.012 ^b^	1.485 ± 0.029 ^d^	1.286 ± 0.0060 ^e^	1.048 ± 0.013 ^g^	0.640 ± 0.004 ^i^	0.709 ± 0.0150 ^h^
Cinnamic acid	0.403 ± 0.002 ^a^	0.175 ± 0.000 ^bc^	0.177 ± 0.005 ^b^	0.172 ± 0.005 ^cd^	0.125 ± 0.004 ^e^	0.168 ± 0.002 ^d^	0.095 ± 0.002 ^h^	0.121 ± 0.001 ^e^	0.107 ± 0.002 ^fg^	0.112 ± 0.001 ^f^	0.110 ± 0.002 ^fg^	0.106 ± 0.002 ^g^
*p*-Hydroxyphenylacetic acid	0.172 ± 0.002 ^i^	0.176 ± 0.001 ^i^	0.508 ± 0.003 ^c^	0.460 ± 0.003 ^d^	0.413 ± 0.002 ^e^	0.364 ± 0.015 ^f^	0.508 ± 0.004 ^c^	0.409 ± 0.004 ^e^	0.273 ± 0.001 ^g^	0.206 ± 0.002 ^h^	2.917 ± 0.024 ^b^	3.098 ± 0.009 ^a^
Phthalic acid	0.364 ± 0.005 ^j^	0.398 ± 0.011 ^i^	1.100 ± 0.001 ^c^	1.154 ± 0.014 ^b^	0.584 ± 0.012 ^g^	1.607 ± 0.016 ^a^	1.069 ± 0.009 ^d^	0.575 ± 0.008 ^g^	0.535 ± 0.003 ^h^	0.396 ± 0.002 ^i^	0.724 ± 0.002 ^f^	0.808 ± 0.006 ^e^
Vanillic acid	0.872 ± 0.005 ^d^	0.704 ± 0.007 ^f^	1.266 ± 0.005 ^b^	1.678 ± 0.013 ^a^	0.318 ± 0.001 ^i^	0.959 ± 0.008 ^c^	0.772 ± 0.008 ^e^	0.321 ± 0.000 ^i^	0.358 ± 0.002 ^h^	0.569 ± 0.014 ^g^	0.258 ± 0.001 ^j^	0.257 ± 0.001 ^j^
Gallic acid	4.774 ± 0.076 ^a^	2.787 ± 0.068 ^e^	2.639 ± 0.003 ^f^	3.151 ± 0.008 ^c^	0.838 ± 0.012 ^j^	1.963 ± 0.001 ^g^	3.209 ± 0.010 ^b^	2.870 ± 0.006 ^d^	1.382 ± 0.022 ^h^	1.342 ± 0.007 ^h^	0.944 ± 0.013 ^i^	0.475 ± 0.004 ^k^
Caffeic acid	1.027 ± 0.004 ^e^	1.085 ± 0.024 ^d^	1.806 ± 0.009 ^c^	2.099 ± 0.019 ^a^	0.372 ± 0.003 ^k^	0.214 ± 0.004 ^l^	1.993 ± 0.004 ^b^	0.392 ± 0.002 ^j^	0.799 ± 0.012 ^h^	1.005 ± 0.005 ^f^	0.765 ± 0.008 ^i^	0.855 ± 0.005 ^g^
Ferulic acid	0.797 ± 0.010 ^e^	0.782 ± 0.011 ^e^	0.830 ± 0.017 ^d^	1.021 ± 0.005 ^c^	0.789 ± 0.002 ^e^	0.707 ± 0.006 ^g^	0.738 ± 0.012 ^f^	1.198 ± 0.016 ^b^	0.471 ± 0.001 ^j^	0.514 ± 0.001 ^i^	0.549 ± 0.000 ^h^	11.861 ± 0.015 ^a^
Sinapic acid	2.692 ± 0.008 ^f^	2.835 ± 0.040 ^e^	3.873 ± 0.010 ^b^	5.255 ± 0.006 ^a^	2.439 ± 0.013 ^g^	3.004 ± 0.010 ^d^	3.807 ± 0.011 ^c^	2.430 ± 0.016 ^g^	1.752 ± 0.025 ^j^	1.856 ± 0.019 ^i^	1.863 ± 0.012 ^i^	2.086 ± 0.014 ^h^
Chlorogenic acid	3.819 ± 0.036 ^f^	4.043 ± 0.064 ^e^	4.358 ± 0.047 ^d^	2.896 ± 0.061 ^g^	0.571 ± 0.005 ^k^	2.233 ± 0.006 ^h^	4.520 ± 0.051 ^c^	0.564 ± 0.021 ^k^	1.481 ± 0.022 ^i^	0.671 ± 0.013 ^j^	5.382 ± 0.074 ^b^	5.680 ± 0.044 ^a^
Naringenin	0.024 ± 0.000 ^i^	0.029 ± 0.001 ^g^	0.033 ± 0.000 ^e^	0.064 ± 0.001 ^c^	0.035 ± 0.000 ^d^	0.033 ± 0.000 ^e^	0.027 ± 0.000 ^h^	0.032 ± 0.000 ^f^	0.019 ± 0.000 ^j^	0.013 ± 0.000 ^k^	0.069 ± 0.000 ^a^	0.067 ± 0.001 ^b^
Eriodictyol	0.013 ± 0.001 ^g^	0.013 ± 0.000 ^g^	0.036 ± 0.001 ^b^	0.067 ± 0.001 ^a^	0.020 ± 0.000 ^d^	0.014 ± 0.000 ^f^	0.030 ± 0.000 ^c^	0.018 ± 0.000 ^e^	0.013 ± 0.000 ^g^	0.005 ± 0.000 ^i^	0.012 ± 0.000 ^h^	0.013 ± 0.000 ^g^
Vitexin	0.178 ± 0.001 ^ab^	0.103 ± 0.003 ^cde^	0.055 ± 0.001 ^e^	0.059 ± 0.001 ^e^	0.146 ± 0.002 ^bc^	0.099 ± 0.001 ^cde^	0.098 ± 0.001 ^cde^	0.143 ± 0.001 ^bc^	0.126 ± 0.001 ^bcd^	0.074 ± 0.003 ^de^	0.150 ± 0.110 ^bc^	0.210 ± 0.002 ^a^
Orientin	0.054 ± 0.000 ^e^	0.020 ± 0.000 ^j^	0.037 ± 0.000 ^i^	0.087 ± 0.004 ^c^	0.045 ± 0.001 ^g^	0.011 ± 0.000 ^k^	0.073 ± 0.001 ^d^	0.049 ± 0.001 ^f^	0.039 ± 0.000 ^h^	0.040 ± 0.001 ^h^	0.113 ± 0.001 ^b^	0.122 ± 0.002 ^a^
Rutin	0.059 ± 0.001 ^j^	0.086 ± 0.002 ^h^	0.080 ± 0.001 ^i^	0.032 ± 0.000 ^k^	0.146 ± 0.002 ^e^	0.103 ± 0.002 ^g^	0.081 ± 0.001 ^i^	0.111 ± 0.003 ^f^	0.180 ± 0.001 ^c^	0.271 ± 0.002 ^a^	0.152 ± 0.000 ^d^	0.263 ± 0.001 ^b^
Paeoniflorin	35.220 ± 0.165 ^i^	39.243 ± 1.839 ^h^	61.551 ± 0.260 ^e^	23.049 ± 0.211 ^j^	63.940 ± 0.160 ^d^	61.463 ± 0.171 ^e^	64.616 ± 0.084 ^d^	60.259 ± 0.204 ^f^	69.270 ± 0.261 ^c^	86.546 ± 0.077 ^a^	41.255 ± 0.087 ^g^	77.222 ± 0.577 ^b^
Albiflorin	6.643 ± 0.013 ^b^	15.300 ± 0.006 ^a^	1.458 ± 0.010 ^g^	0.682 ± 0.001 ^i^	1.526 ± 0.016 ^f^	1.458 ± 0.021 ^g^	1.551 ± 0.008 ^f^	1.452 ± 0.033 ^g^	1.896 ± 0.019 ^e^	2.267 ± 0.012 ^c^	1.115 ± 0.010 ^h^	2.109 ± 0.019 ^d^
Oxypaeoniflorin	0.608 ± 0.010 ^k^	0.765 ± 0.008 ^j^	0.809 ± 0.003 ^i^	0.313 ± 0.003 ^l^	1.469 ± 0.020 ^e^	1.162 ± 0.022 ^g^	1.021 ± 0.028 ^h^	1.217 ± 0.005 ^f^	1.892 ± 0.030 ^c^	3.329 ± 0.005 ^a^	1.502 ± 0.010 ^d^	3.188 ± 0.011 ^b^
Mudanpioside D	0.569 ± 0.011 ^h^	0.581 ± 0.0098 ^h^	1.753 ± 0.004 ^c^	2.444 ± 0.018 ^b^	1.213 ± 0.004 ^f^	3.149 ± 0.013 ^a^	1.746 ± 0.014 ^c^	1.181 ± 0.012 ^g^	1.167 ± 0.014 ^g^	1.374 ± 0.010 ^e^	1.381 ± 0.025 ^e^	1.663 ± 0.014 ^d^
Mudanpioside E	0.368 ± 0.003 ^g^	0.438 ± 0.002 ^e^	0.474 ± 0.002 ^d^	0.479 ± 0.006 ^d^	0.359 ± 0.002 ^h^	0.480 ± 0.003 ^d^	0.495 ± 0.002 ^c^	0.338 ± 0.002 ^i^	0.431 ± 0.003 ^f^	0.424 ± 0.003 ^f^	0.715 ± 0.010 ^b^	0.886 ± 0.006 ^a^
Benzoylpaeoniflorin	0.303 ± 0.018 ^d^	0.347 ± 0.001 ^b^	0.223 ± 0.002 ^i^	0.267 ± 0.009 ^f^	0.587 ± 0.001 ^a^	0.251 ± 0.002 ^h^	0.258 ± 0.001 ^g^	0.181 ± 0.012 ^j^	0.189 ± 0.005 ^j^	0.189 ± 0.000 ^j^	0.286 ± 0.000 ^e^	0.331 ± 0.001 ^c^
Benzoyloxypaeoniflorin	0.050 ± 0.000 ^h^	0.068 ± 0.007 ^gh^	0.063 ± 0.000 ^gh^	0.065 ± 0.000 ^gh^	0.161 ± 0.002 ^d^	0.082 ± 0.002 ^fgh^	0.103 ± 0.001 ^efg^	0.131 ± 0.001 ^de^	0.218 ± 0.002 ^c^	0.118 ± 0.088 ^def^	0.417 ± 0.001 ^b^	0.652 ± 0.003 ^a^
Mudanpioside C	0.246 ± 0.003 ^a^	0.234 ± 0.001 ^b^	0.110 ± 0.001 ^f^	0.050 ± 0.000 ^k^	0.088 ± 0.001 ^h^	0.198 ± 0.001 ^c^	0.072 ± 0.003 ^j^	0.074 ± 0.002 ^i^	0.104 ± 0.001 ^g^	0.149 ± 0.000 ^e^	0.160 ± 0.003 ^d^	0.238 ± 0.007 ^b^
Mudanpioside A	0.275 ± 0.008 ^f^	0.190 ± 0.002 ^i^	0.357 ± 0.001 ^d^	0.496 ± 0.001 ^a^	0.216 ± 0.003 ^g^	0.396 ± 0.001 ^c^	0.345 ± 0.003 ^e^	0.202 ± 0.002 ^h^	0.077 ± 0.001 ^k^	0.180 ± 0.002 ^j^	0.394 ± 0.003 ^c^	0.429 ± 0.001 ^b^
Mudanpioside H	0.092 ± 0.001 ^i^	0.103 ± 0.005 ^h^	0.089 ± 0.003 ^i^	0.083 ± 0.007 ^j^	0.211 ± 0.002 ^e^	0.131 ± 0.007 ^g^	0.181 ± 0.002 ^f^	0.174 ± 0.004 ^f^	0.275 ± 0.000 ^d^	0.394 ± 0.000 ^b^	0.367 ± 0.003 ^c^	0.509 ± 0.001 ^a^
Mudanpioside J	0.660 ± 0.007 ^j^	0.702 ± 0.015 ^i^	1.241 ± 0.002 ^f^	1.747 ± 0.003 ^d^	0.821 ± 0.021 ^g^	1.442 ± 0.015 ^e^	1.230 ± 0.008 ^f^	0.817 ± 0.021 ^g^	0.750 ± 0.002 ^h^	2.452 ± 0.024 ^a^	1.951 ± 0.010 ^c^	2.230 ± 0.005 ^b^
Galloylpaeoniflorin	0.133 ± 0.003 ^j^	0.185 ± 0.000 ^g^	0.166 ± 0.002 ^h^	0.055 ± 0.000 ^k^	0.260 ± 0.001 ^d^	0.216 ± 0.003 ^f^	0.153 ± 0.002 ^i^	0.217 ± 0.003 ^f^	0.275 ± 0.000 ^c^	0.444 ± 0.003 ^a^	0.252 ± 0.012 ^e^	0.368 ± 0.002 ^b^
Galloyloxypaeoniflorin	0.117 ± 0.010 ^j^	0.147 ± 0.003 ^h^	0.190 ± 0.001 ^fg^	0.278 ± 0.001 ^d^	0.125 ± 0.003 ^i^	0.196 ± 0.003 ^f^	0.189 ± 0.002 ^g^	0.124 ± 0.003 ^i^	0.247 ± 0.002 ^e^	0.525 ± 0.001 ^a^	0.385 ± 0.002 ^c^	0.446 ± 0.000 ^b^
paeonol	1.300 ± 0.019 ^j^	1.449 ± 0.056 ^i^	4.034 ± 0.004 ^c^	4.201 ± 0.049 ^b^	2.128 ± 0.044 ^g^	5.841 ± 0.057 ^a^	3.914 ± 0.031 ^d^	2.094 ± 0.028 ^g^	1.902 ± 0.048 ^h^	1.425 ± 0.006 ^i^	2.586 ± 0.007 ^f^	2.904 ± 0.021 ^e^
Mudanoside A	5.021 ± 0.048 ^d^	5.545 ± 0.131 ^c^	7.093 ± 0.030 ^b^	1.553 ± 0.033 ^k^	4.718 ± 0.066 ^e^	7.353 ± 0.046 ^a^	4.594 ± 0.006 ^f^	4.235 ± 0.009 ^g^	4.111 ± 0.013 ^h^	2.303 ± 0.002 ^i^	1.975 ± 0.037 ^j^	2.361 ± 0.007 ^i^
Mudanoside B	1.032 ± 0.031 ^d^	1.263 ± 0.141 ^c^	1.822 ± 0.018 ^a^	0.842 ± 0.003 ^e^	1.088 ± 0.004 ^d^	0.625 ± 0.003 ^g^	1.575 ± 0.056 ^b^	1.034 ± 0.005 ^d^	0.828 ± 0.005 ^e^	1.020 ± 0.004 ^d^	0.747 ± 0.003 ^f^	0.846 ± 0.002 ^e^
Suffruticoside A or C	0.043 ± 0.000 ^h^	0.053 ± 0.001 ^g^	0.057 ± 0.000 ^f^	0.019 ± 0.000 ^j^	0.183 ± 0.005 ^b^	0.107 ± 0.000 ^d^	0.079 ± 0.002 ^e^	0.146 ± 0.004 ^c^	0.210 ± 0.001 ^a^	0.032 ± 0.000 ^i^	0.058 ± 0.000 ^f^	0.058 ± 0.000 ^f^
Oxyresveratrol	0.041 ± 0.000 ^a^	0.041 ± 0.000 ^a^	0.040 ± 0.000 ^b^	0.019 ± 0.000 ^h^	0.040 ± 0.000 ^e^	0.019 ± 0.000 ^i^	0.009 ± 0.000 ^j^	0.040 ± 0.000 ^d^	0.001 ± 0.000 ^e^	0.008 ± 0.000 ^k^	0.037 ± 0.000 ^g^	0.038 ± 0.000 ^f^

^1^ Values are means standard deviations, *n* = 3. Different superscript letters within the same row indicate significant differences (one-way ANOVA and Duncan test, *p* extra).

## Supplementary Materials

The following are available online at https://www.mdpi.com/2304-8158/9/6/770/s1, Table S1: Oil content and relative fatty acid composition (%) of ten *Paeonia* seed oil samples.
