# Supplementary material for: Integrated Profiling of Fatty Acids, Sterols and Phenolic Compounds in Tree and Herbaceous Peony Seed Oils: Marker Screening for New Resources of Vegetable Oil
Source: Foods. 2020 Jun 11;9(6):770. doi: 10.3390/foods9060770 (PMC7353516; doi:10.3390/foods9060770)
Supplement: Supplementary file 1 [file foods-09-00770-s001.pdf]

Integrated profiling of fatty acids, sterols and phenolic compounds in tree and herbaceous peony seed oils: Marker screening for new resources of vegetable oil

Xiaoqin Wang [1,2,*], Chunhuan Li [1,2,3], María del Mar Contreras [4*], Vito Verardo [5,6], Ana María Gómez-Caravaca [7], Chen Xing [1,2]

[1] College of Chemical Engineering, Huaqiao University, Xiamen, Fujian 361021, China; xiaoqin77@foxmail.com (X.W.); 1192535487@qq.com (C.L.); xingchen94621@qq.com (C.X.)

[2] Institute of Oil and Natural Product, Huaqiao University, Xiamen, Fujian 361021, China

[3] Shandong Institute for Food and Drug Control, Jinan, Shandong 250101, China

[4] Department of Chemical, Environmental and Materials Engineering, Universidad de Jaén, Campus Las Lagunillas, Jaén 23071, Spain; mcgamez@ujaen.es

[5] Department of Nutrition and Food Science, University of Granada, Campus of Cartuja, 18071, Granada, Spain; vitoverardo@ugr.es

[6] Institute of Nutrition and Food Technology 'José Mataix', Biomedical Research Centre, University of Granada, Avenida del Conocimiento s/n, E-18071 Granada, Spain

[7] Department of Analytical Chemistry, University of Granada, c/Fuentenueva s/n, E-18071 Granada, Spain; anagomez@ugr.es

* Correspondence: mcgamez@ujaen.es (mar.contreras.gamez@gmail.com or mmcontreras@ugr.es); Tel. +34 953 21 27 99 (M.d.M.C); xiaoqin77@foxmail.com; Tel.: 86-0592-6166087 (X.W.)

Supplementary information

Table S1. Oil content and relative fatty acid composition (%) of ten *Paeonia* seed oil samples [1].

**Cultivar/Species**	*P. ostii*									*P. rockii*	**TuoPanXianBao**	**LianTai**
**Region**	**SXWN**	**SXSL**	**SDHZ**	**HNLY**	**AHTL**	**AHBZ**	**JSSY**	**HBWH**	**GSDX**	**GSLZ**	**SDHZ**	**SDHZ**
Oil yield (%)	33.64± 0.19^{a}	32.96± 0.26^{b}	30.65± 0.10^{e}	31.83± 0.10^{c}	34.00± 0.26^{a}	30.67± 0.32^{e}	27.70± 0.58^{g}	31.06± 0.49^{de}	31.70± 0.45^{cd}	29.39± 0.53^{f}	32.17± 0.16^{c}	26.86± 0.12^{h}
C14:0	0.0554± 0.0001^{f}	0.0613± 0.0001^{a}	0.0547± 0.0001^{g}	0.0453± 0.0001^{k}	0.0549± 0.0002^{g}	0.0528± 0.0001^{h}	0.0602± 0.0001^{c}	0.0602± 0.0001^{c}	0.0606± 0.0001^{b}	0.0568± 0.0001^{e}	0.0577± 0.0001^{d}	0.0479± 0.0002^{j}
C15:0	0.0353± 0.0001^{b}	0.0339± 0.0001^{d}	0.0332± 0.0001^{e}	0.0338± 0.0001^{d}	0.0345± 0.0001^{c}	0.0335± 0.0003^{e}	0.0309± 0.0003^{f}	0.0307± 0.0002^{f}	0.0361± 0.0001^{a}	0.0256± 0.0001^{h}	0.0302± 0.0002^{g}	0.0241± 0.0001^{j}
C16:0	5.7978± 0.0029^{c}	5.8844± 0.0026^{a}	5.5320± 0.0049^{h}	5.3379± 0.0031^{j}	5.7301± 0.0011^{d}	5.5478± 0.0047^{g}	5.6982± 0.0024^{e}	5.6905± 0.0011^{f}	5.8195± 0.0007^{b}	4.9419± 0.0010^{k}	5.4958± 0.0019^{i}	3.8477± 0.0030^{l}
C16:1	0.1001± 0.0004^{d}	0.0922± 0.0002^{i}	0.0968± 0.0002^{f}	0.0896± 0.0003^{j}	0.0954± 0.0001^{g}	0.0934± 0.0010^{h}	0.1041± 0.0004^{b}	0.1038± 0.0005^{b}	0.1028± 0.0004^{c}	0.1049± 0.0001^{a}	0.0984± 0.0003^{e}	0.0660± 0.0001^{k}
C17:0	0.1313± 0.0005^{b}	0.1142± 0.0002^{d}	0.1113± 0.0003^{f}	0.1112± 0.0004^{f}	0.1121± 0.0001^{e}	0.1209± 0.0003^{c}	0.1002± 0.0006^{h}	0.0997± 0.0009^{h}	0.1320± 0.0006^{b}	0.1378± 0.0001^{a}	0.1012± 0.0005^{g}	0.0696± 0.0001^{i}
C17:1	0.0929± 0.0001^{d}	0.0705± 0.0005^{j}	0.0830± 0.0002^{f}	0.0819± 0.0003^{g}	0.0793± 0.0001^{i}	0.0909± 0.0007^{e}	0.0809± 0.0007^{h}	0.0806± 0.0005^{h}	0.0971± 0.0005^{c}	0.1333± 0.0001^{b}	0.0790± 0.0001^{i}	0.1388± 0.0006^{a}
C18:0	1.9725± 0.0016^{b}	2.2607± 0.0014^{a}	1.7420± 0.0009^{i}	1.7767± 0.0011^{g}	1.8121± 0.0024^{f}	1.7507± 0.0005^{h}	1.8610± 0.0009^{d}	1.8511± 0.0020^{e}	1.8695± 0.0009^{c}	1.6481± 0.0012^{j}	1.8716± 0.0004^{c}	0.8142± 0.0012^{k}
C18:1n9	21.7568± 0.0055^{h}	21.4387± 0.0005^{i}	21.8905± 0.0139^{g}	20.6338± 0.0085^{l}	22.0926± 0.0061^{f}	21.0147± 0.0069^{k}	23.8147± 0.0031^{d}	23.7933± 0.0016^{e}	21.3418± 0.0044^{j}	26.7798± 0.0011^{b}	23.8279± 0.0055^{c}	31.3515± 0.0101^{a}
C18:1n11	0.3355± 0.0018^{d}	0.3280± 0.0012^{ef}	0.3478± 0.0028^{bc}	0.3247± 0.0034^{f}	0.3313± 0.0027^{de}	0.3170± 0.0069^{g}	0.3523± 0.0006^{b}	0.3522± 0.0023^{b}	0.3265± 0.0022^{ef}	0.2994± 0.0019^{h}	0.3458± 0.0015^{c}	0.4127± 0.0069^{a}
C18:2n6	24.6044± 0.0029^{h}	23.9758± 0.0011^{i}	25.7201± 0.0029^{e}	27.7490± 0.0095^{c}	29.9448± 0.0029^{b}	27.6882± 0.0046^{d}	25.0237± 0.0008^{g}	25.0396± 0.0191^{f}	23.2454± 0.0139^{k}	16.9078± 0.0025^{l}	23.6166± 0.0014^{j}	30.7427± 0.0054^{a}

α--C18:3 n3	0.2089± 0.0024^{de}	0.2192± 0.0014^{b}	0.2261± 0.0048^{a}	0.2191± 0.0006^{b}	0.1939± 0.0021^{g}	0.2115± 0.0002^{cd}	0.2077± 0.0009^{ef}	0.2047± 0.0014^{f}	0.2147± 0.0014^{c}	0.1930± 0.0007^{g}	0.2077± 0.0016^{ef}	0.1617± 0.0009^{h}

[1] Values are means ± standard deviations, n=3. Different superscript letters within the same row indicate significant differences (one-way ANOVA and Duncan test, $p \leq 0.05$).

SFA, saturated fatty acids; MUFA, monounsaturated fatty acids; PUFA, polyunsaturated fatty acids; UFA, unsaturated fatty acids.
